# Association between bullying victimization and health risk behavior in adolescents

**DOI:** 10.1590/1984-0462/2025/43/2023215

**Published:** 2024-09-06

**Authors:** Ana Beatriz Pacífico, Eliane Denise Araújo Bacil, Mariana Ardengue, Thiago Silva Piola, Michael Pereira da Silva, Fabio Fontana, Ademar Avelar, Wagner de Campos

**Affiliations:** a Universidade Federal do Paraná, Curitiba, PR, Brazil.; b Universidade Estadual de Maringá, Maringá, PR, Brazil.; c Universidade Federal de Rio Grande, Rio Grande, RS, Brazil.; d University of Northern Iowa, Cedar Falls, Iowa, United States.

**Keywords:** Bullying, Physical activity, Sedentary behavior, Health risk behaviors, Adolescents, Bullying, Atividade física, Comportamento sedentário, Comportamentos de risco à saúde, Adolescentes

## Abstract

**Objective:**

The aim of this study was to examine the association between bullying victimization and health risk behaviors in adolescents.

**Methods:**

A representative sample of 1020 adolescents participated in the study. The variables such as bullying, health risk behaviors (tobacco, drugs, alcohol, sedentary behavior, smartphone use, level of physical activity, and sleep), and economic status were assessed using self-reported questionnaires. Odds ratios with 95% confidence intervals (95%CI) were obtained using binary logistic regression and ordinal, gross, and adjusted logistic regression (p<0.05).

**Results:**

Victims of bullying were more likely to smoke (OR 1.75; 95%CI 1.28–2.40), consume alcohol (OR1.43; 95%CI 1.05–1.94), have worse sleep quality (OR 1.94; 95%CI 1.28–2.91), and more sedentary behavior (OR 1.43; 95%CI 1.08–1.89) than those who were not bullied. However, victims were more likely to have high levels of physical activity than their non-bullied peers (OR 1.66; 95%CI 1.22–2.27).

**Conclusions:**

Bullying victimization was associated with an increased predisposition for the adoption of health risk behaviors. Interestingly, victims were also more prone to participate in physical activity.

## RESUMO

**Objetivo:**

Examinar a associação entre vitimização por *bullying* e comportamentos de risco à saúde em adolescentes.

**Métodos:**

Uma amostra representativa de 1.020 adolescentes participou do estudo. As variáveis *bullying*, comportamentos de risco à saúde (tabaco, drogas, álcool, comportamento sedentário, uso de *smartphone*, nível de atividade física, sono) e situação econômica foram avaliadas por meio de questionários autorreportados. Razões de chance com intervalos de confiança de 95% (IC95%) foram obtidas por meio de regressão logística binária e regressão logística ordinal, bruta e ajustada (p<0,05).

**Resultados:**

As vítimas de *bullying* apresentaram maior probabilidade de fumar (OR 1,75; IC95% 1,28–2,40), consumir álcool (OR 1,43; IC95% 1,05–1,94), ter pior qualidade de sono (OR 1,94; IC95% 1,28–2,91) e apresentar mais comportamento sedentário (OR 1,43; IC95% 1,08–1,89) do que aqueles que não sofreram *bullying*. No entanto, as vítimas eram mais propensas a ter níveis elevados de atividade física do que os seus pares não vítimas de *bullying* (OR 1,66; IC95% 1,22–2,27).

**Conclusões:**

A vitimização por *bullying* esteve associada ao aumento da predisposição para a adoção de comportamentos de risco à saúde. Curiosamente, as vítimas também eram mais propensas a participar de atividades físicas.

## INTRODUCTION

Adolescence is a stage of development characterized by several biological, behavioral, and psychosocial changes. It may be a troubled and confusing developmental period in which contradictory and inconsistent feelings may turn into aggressive behaviors such as bullying.^1^ Bullying is a repetitive act of physical or verbal violence, which is a highly prevalent behavior during adolescence worldwide.^2^ Bullying is also prevalent in Brazil. For example, according to PeNSE 2019, 23% of school age adolescents in Brazil (13–17 years of age) reported feeling humiliated by colleagues in the past 30 days.^3^ Bullying is a serious public health problem^3^ and can negatively affect adolescents. According to the Centers for Disease Control and Prevention (CDC), young people who are victims of bullying tend to experience loneliness, anxiety, depression, sleep difficulties, poor school performance,^4^ and are also more likely to commit suicide.^5^

Victimization may also lead to health risk behaviors (HRBs) such as sedentary behavior, insufficient levels of physical activity, insufficient sleep, and the use of tobacco, illicit drugs, and alcohol.^6,7,8^ Unfortunately, the literature examining the association between bullying and HRBs is limited, and the findings are not consistent across studies. The literature examining the association between bullying and HRB is even scarcer in Brazil. In addition, many existing studies have methodological weaknesses, including the use of non-validated questionnaires for the assessment of bullying and non-representative participant samples. Thus, this study aims to examine the association between bullying victimization and HRBs in male and female adolescents in Maringá (PR). We hypothesized that adolescents reporting victimization are more likely to engage in HRB.

## METHOD

This is a cross-sectional study with a representative sample of adolescents enrolled in public schools in Maringá (PR). Maringá is a medium- to large-sized urban center. It has the third largest population in the state, with approximately 436,472 inhabitants. The study adopted resolution CNS 466/2012 of the National Health Council of the Ministry of Health, which approved the “New Guidelines and Regulatory Norms for Research Involving Human Beings” (DOU 1996 Oct 16; no201, section 1:21082-21085). The study was approved by the Research Ethics Committee of Unicesumar University, CAAE: 57872522.6.0000.5539. Data collection was carried out in 2022 from April to June by a trained team. The data collection took place shortly after schools returned to in-person classes after a period of social isolation caused by the COVID-19 pandemic. The target population was middle-school students attending public schools in Maringá (PR). Based on IBGE statistics, the total number of students was 12,130.^9^

The sample consisted of adolescents from both sexes aged 15–17 years. Sample size calculations were performed using the Cochran-Mantel-Haenszel^10^ method for analysis of the association between dichotomous variables. All calculations were performed considering a significance level of 5%, a power of 80%, and a 1:1 ratio of exposed to unexposed. Bullying was considered the main predictor variable. The study outcomes were smoking, alcohol consumption, illicit drug use, poor sleep quality, insufficient practice of physical activity, and sedentary behavior. A total of six sample size calculations were carried out. For each calculation, the prevalence of the outcome in each exposure group was considered to be 50%, a default value when there is insufficient information about the prevalence of outcomes in the exposure groups. The estimated ORs were obtained from previous literature studies (supplementary document). The sample size required for the study ranged from 172 (illicit drug use) to 776 individuals (sedentary behavior). Due to possible losses, 30% was added to the highest estimate so that the minimum estimated sample size was 1009 adolescents.

Based on the division of neighborhoods in Maringá (PR), a single school from each region (north, south, east, west, and central) was randomly selected according to student enrollment.

Thus, the sample was selected in a probabilistic way in three stages:

1. All public schools were listed and stratified according to the five city regions;
2. A school was randomly selected from each region;
3. A simple random selection of classrooms from each school was performed.

The number of classrooms depended on the number of students necessary to accurately represent each region. A total of 1,308 adolescents were assessed. The final sample was made up of 1,020 adolescents after the exclusion of participants according to the following criteria: age under 15 or over 17 years, reporting a disability, and questionnaires with missing data.

The Olweus Bullying Questionnaire (QBO),^11^ a Brazilian version of the Revised Olweus Bully/Victim Questionnaire (OBVQ),^12^ was used to assess and classify victims of bullying. This instrument contains 23 items inquiring about the frequency in which individuals have experienced and/or engaged in bullying behavior. Those who answered that they were bullied “once or more times a week” were classified as victims of bullying. The internal reliability of the victim scale was adequate (Cronbach’s α=0.85).

Sedentary behavior was assessed using the Brazilian version^13^ of the Sedentary Activities Questionnaire (QASA) for adolescents.^14^ This instrument provides information on the time spent in hours and/or minutes on different types of sedentary activities during week and weekend days of a typical week. The classification was given by the tertile of hours presented by the sample: “High,” “Medium,” and “Low” time in sedentary behavior. The validity of QASA to measure the sedentary behaviors of Brazilian adolescents is adequate. For example, the test–retest weekday ICC was 0.88 (95%CI 0.82–0.91), and the test–retest weekend ICC was 0.77 (95%CI 0.68–0.84).

The Short Version of the Smartphone Addiction Scale (SAS-SV)^15^ was used to verify whether adolescents are classified as addicted to smartphone use. This questionnaire consists of 10 questions about the use of phone devices, with response options ranging from 1 to 6 on a Likert scale from “Totally disagree” to “Totally agree.” Total scores may vary from 10 to 60 points. The authors suggested a cutoff point of 33. Those with a score greater than 33 are considered to have a smartphone addiction.^15^ SAS-SV has adequate reliability for assessing smartphone addiction among Brazilian adolescents (α=0.81; ω=0.78).

The Brazilian version of the *Physical Activity Questionnaire for Adolescents* (PAQ-A)^16^ was used to assess insufficient levels of physical activity. This questionnaire is aimed at adolescents aged between 14 and 18 years. It measures engagement in physical activity in the last 7 days through 8 Likert items ranging from 1 to 5. More specifically, the PAQ-A gathers information regarding the frequency and intensity of physical activity during free time and physical education classes. Adolescents were classified as less and more active using the median of the sample as the cutoff point. The PAQ-A has positive indicators of validity and test–retest reliability, with ICCs ranging between 0.68 and 0.88 and an internal consistency of α=0.76.

The Brazilian version of the Pittsburgh Sleep Quality Index (PSQI)^17^ was used to assess sleep time and quality. Individuals with less than 8 h of sleep per day (during the week and weekends) were classified as having inadequate sleep time, while those with more than 8 h of sleep per day were classified as having adequate sleep time.^18^ The questionnaire assessed seven components of sleep quality: subjective quality, sleep latency, sleep duration, sleep efficiency, sleep disturbances, medication use, and daily dysfunction. Scores varied from 0 to 3 for each component. The maximum total sleep quality score was 21 points, with higher scores representing worse sleep quality. Adolescents with five or more points were classified as having poor sleep quality. The PSQI was translated and validated to assess sleep time and quality among Brazilian adolescents. It has adequate internal consistency (α=0.82) and test–retest reliability.

The Brazilian version^19^ of the *Youth Risk Behavior Survey* (YRBS) was used to assess the consumption of cigarettes, illicit drugs, and alcohol. YRBS was initially developed by the *Center for Disease Control and Prevention.* ^20^ The following were considered risk factors: consuming at least one cigarette, some type of illicit drug (at least once), and at least one dose of alcohol in the last 30 days before data collection. The mean Kappa concordance was 68.6% indicating the quality of psychometric properties of the Portuguese version.

The control variables in this study were sex, age group (divided by the age of 15, 16, and 17 years), socioeconomic class, and weight status. Sex and age range were obtained through anamnesis. The assessment of socioeconomic class was carried out using the Brazil Economic Classification Criteria Questionnaire, proposed by the Brazilian Association of Research Companies.^21^ This criterion estimates the purchasing power of families based on the availability of home appliances and the level of education of the head of the household. For this study, the sample was classified into high (class A), medium (classes B1 and B2), and low (classes C and D) socioeconomic classes.

Absolute and relative frequencies were used to describe sociodemographic characteristics and the outcome and predictor variables. The chi-square test was used to compare proportions and possible differences between sexes. For the analysis of the association between bullying and HRBs (insufficient levels of physical activity, insufficient sleep, sleep quality, use of tobacco, illicit drugs, and alcohol), the binary logistic regression procedure was used for dichotomous outcomes, and the ordinal logistic regression was used for the ordinal outcome (sedentary behavior). The control variables used during adjusted analyses were nutritional status, sex, age, and economic class. The analyses were performed using the Stata statistical software version 15.0 (StataCorp LLC, College Station, TX, USA) with a significance level of p≤0.05.

## RESULTS

The final sample consisted of 1020 adolescents evenly distributed by sex (Male=50.1%). Table 1 shows the description of exposure variables, outcome, and covariates. Approximately 20% of the sample reported being a victim of bullying. Victimization was not significantly different between the sexes. A large proportion of the sample (75%) reported poor sleep quality. The proportion of females (83.3%) with poor sleep quality was significantly larger than that of males (68.3%, p<0.001). Most of the adolescents (72.5%) reported sleeping less than 8 h/day, and sleep duration was not different between sexes. Notably, 31% of the sample reported smoking within the past 30 days, and 38.5% reported alcohol consumption. More females reported smoking (35.4%) and alcohol consumption (42%) than males (31.1% and 35%, respectively). The proportion of girls (59.1%) classified as less active was significantly larger than that of boys (40.9%, p<0.001). The proportion of adolescents classified as addicted to smartphones was 34.4%. A significantly larger proportion of girls (43.6%) reported smartphone addiction than boys (25.2%, p<0.001).

**Table 1:** Descriptive characteristics of participants (n=1020).

	Total	Male	Female	χ^2^	p-value

	n (%)	n (%)	n (%)		
Age (years)					
15	257 (25.1)	128 (25.1)	128 (25.2)	2.99	0.393
16	362 (35.5)	172 (33.7)	190 (37.4)		
17	401 (39.4)	211 (41.2)	190 (37.4)		
Economic class					
High	340 (33.3)	168 (32.9)	172 (33.8)	0.14	0.933
Middle	567 (55.6)	287 (56.1)	280 (55.0)		
Low	113 (11.1)	56 (11.0)	57 (11.2)		
Bullying-victim					
No	815 (79.9)	415 (81.2)	400 (78.6)	1.10	0.295
Yes	205 (20.1)	96 (18.8)	109 (21.4)		
Sleep quality					
Good	247 (24.2)	162 (31.7)	85 (16.7)	31.28	**<0.001**
Bad	773 (75.8)	349 (68.3)	424 (83.3)		
Hours of sleep per day					
<8	740 (72.5)	358 (70.1)	382 (75.0)	3.19	0.074
≥8	280 (27.5)	153 (29.9)	127 (25.0)		
Smoking in the last 30 days					
No	696 (68.2)	367 (71.8)	329 (64.6)	6.07	**0.014**
Yes	324 (31.8)	144 (28.2)	180 (35.4)		
Alcohol consumption					
No	627 (61.5)	332 (65.0)	295 (58.0)	5.30	**0.021**
Yes	393 (38.5)	179 (35.0)	214 (42.0)		
Use of illicit drugs in the last 30 days					
No	970 (95.1)	477 (93.3)	493 (96.9)	6.74	**0.009**
Yes	50 (4.9)	34 (6.7)	15 (3.1)		
Physical activity*					
Less active	510 (50.0)	209 (40.9)	301 (59.1)	33.92	**<0.001**
More active	510 (50.0)	302 (59.1)	208 (40.9)		
Smartphone addiction					
No	669 (65.6)	382 (74.8)	287 (56.4)	38.13	**<0.001**
Yes	351 (34.4)	129 (25.2)	222 (43.6)		
Sedentary behavior					
Low sedentary time	341 (33.4)	179 (35.0)	162 (31.8)	2.41	0.299
Medium sedentary time	339 (33.3)	173 (33.9)	166 (32.6)		
High sedentary time	340 (33.3)	159 (31.1)	181 (35.6)		

χ^2^: chi-square test; p≤0.05.

*Classification obtained by the median scores: median (IQR)=2.19 (1.67–2.77). Bold denotes statistically significant p-values.

Table 2 shows the association between HRBs and being a victim of bullying. Adolescent victims of bullying were 1.75 times more likely to smoke (95%CI 1.28–2.40), 1.43 times more likely to consume alcohol (95%CI 1.05–1.94), 1.94 times more likely to have worse sleep quality (95%CI 1.28–2.91), and 1.66 times more likely to do more physical activity than those who were not victims of bullying (95%CI 1.22–2.27).

**Table 2:** Associations between victimization and health risk behaviors.

	Bullying-victim		

	Total	Male	Female

	OR (95%CI)	OR (95%CI)	OR (95%CI)
Smoking	1.75 (1.28–2.40)	1.81 (1.13–2.88)	1.68 (1.09–2.58)
Alcohol consumption	1.43 (1.05–1.94)	1.49 (0.95–2.35)	1.34 (0.88–2.05)
Use of illicit drugs	1.13 (0.57–2.24)	1.13 (0.48–2.68)	1.23 (0.39–3.90)
Smartphone addiction	1.25 (0.91–1.72)	1.54 (0.95–2.50)	1.02 (0.67–1.56)
Sleep quality	1.94 (1.28–2.91)	1.98 (1.16–3.37)	1.80 (0.94–3.46)
Hours of sleep per day	1.02 (0.73–1.44)	1.15 (0.71–1.85)	0.93 (0.56–1.52)
Physical activity	1.66 (1.22–2.27)	2.41 (1.46–3.98)	1.36 (0.89–2.08)
Sedentary behavior*	1.43 (1.08–1.89)	1.18 (0.79–1.76)	1.69 (1.14–2.51)

*Ordinal logistic regression model for ordinal polytomous outcomes.

When stratified by sex, boys who were victims of bullying were 1.81 times more likely to smoke (95%CI 1.13–2.88), 1.98 times more likely to have poorer sleep quality (95%CI 1.16–3.37), and 2.41 times more likely to engage in physical activity (95%CI 1.46–3.98). Girls reporting victimization were 1.68 times more likely to smoke compared to those who were not victims of bullying (95%CI 1.09–2.58).

Finally, being a victim of bullying increased the chance of switching sedentary behavior categories from low to medium or medium to high by 43% (OR 1.43; 95%CI 1.08–1.89). When stratified by sex, girls were 69% more likely to switch sedentary behavior categories from low to medium or medium to high (OR 1.69; 95%CI 1.14–2.51).

Table 3 shows the adjusted associations between HRBs and being a victim of bullying. The unadjusted analysis using the total sample produced very similar results to the analysis adjusted by sex, socioeconomic level, and age. When stratified by sex and adjusted by age and socioeconomic status, boys reporting being victims of bullying had a 1.54 times greater chance of consuming alcohol compared to nonvictims (95%CI 1.10–1.73).

**Table 3:** Adjusted associations between health risk behaviors and victimization.

	Bullying-victim		

	Total*	Male^†^	Female^†^

	OR (95%CI)	OR (95%CI)	OR (95%CI)
Smoking	1.75 (1.28–2.41)	1.83 (1.15–2.92)	1.68 (1.09–2.60)
Alcohol consumption	1.45 (1.06–1.98)	1.54 (1.10–1.73)	1.37 (0.88–2.12)
Use of illicit drugs	1.18 (0.59–2.37)	1.16 (0.49–2.76)	1.20 (0.38–3.86)
Smartphone addiction	1.21 (0.87–1.67)	1.55 (0.95–2.52)	1.00 (0.65–1.53)
Sleep quality	1.91 (1.26–2.88)	1.99 (1.17–3.39)	1.77 (0.92–3.39)
Hours of sleep per day	1.03 (0.73–1.45)	1.13 (0.69–1.83)	0.95 (0.57–1.55)
Physical activity	1.75 (1.27–2.40)	2.44 (1.47–4.03)	1.35 (0.88–2.07)
Sedentary behavior	1.42 (1.07–1.88)	1.21 (0.81–1.81)	1.70 (1.14–2.52)

*Adjusted for sex, age, and socioeconomic status;

^†^Adjusted for age and socioeconomic status.

## DISCUSSION

This study showed that a high number of adolescents are victims of bullying in schools. In fact, 20.1% of participants reported bullying victimization. This value is higher when compared to the national survey by PeNSE (2015),^22^ which showed that 7.4% of Brazilian adolescents had been bullied. However, the number is closer to the average prevalence of 35% of bullying victimization worldwide.^23^

Besides that, this study indicated that being bullied is associated with HRBs among adolescents. Victims of bullying were at greater risk for smoking, consuming alcohol, having worse sleep quality, and engaging in more sedentary behavior than those who were not victims of bullying. The only exception was physical activity. Victims of bullying reported practicing more physical activity than adolescents who reported not being victims of bullying. As detailed in-depth below, these behaviors are associated with significant short- and long-term health risks for adolescents.

Victimization, independent of sex, was associated with a higher likelihood of smoking during adolescence. This finding is supported by other studies.^24^ Victims may take refuge in smoking addiction or other actions to fit in with their peers. The habit of smoking is often established during adolescence. In fact, most smokers first try or become addicted to tobacco before the age of 18 years. This association can have extremely negative long-term consequences for adolescents. In fact, smoking is the main cause of death for about half of those who continue to smoke after adolescence.^25^ Public policy to prevent bullying may inadvertently reduce adolescent smoking.

Victimization was also shown to be associated with alcohol consumption in the analysis combining boys and girls. When stratified by sex, this association remained significant for boys only. The results are in agreement with previous studies showing an association between being a victim of bullying and alcohol consumption in both sexes.^24,26^ Alcohol consumption in adolescence tends to be associated with a series of negative outcomes such as low academic performance, higher incidence of car accidents, higher levels of engagement violent behavior, and higher consumption of other harmful substances such as tobacco and illicit drugs.^26^ Similar to smoking, alcohol consumption may be a way for adolescents to deal with stress or improve their sense of belonging to peers.

Additionally, the analysis using the total sample indicated that victims of bullying were more likely to report poor sleep quality. When stratified by sex, this association remained significant for boys only. Other studies have also demonstrated an association between sleep and victimization.^27^ Sleep problem is another health outcome that should be added to the array of negative consequences of bullying for adolescents. Poor sleep quality is associated with worse perceptions of health in general and, more specifically, psychosomatic health complaints, including headache, back pain, irritability, bad mood, nervousness, and dizziness.^28^

Sedentary behavior was also associated with victimization in the analysis combining boys and girls. This association was present only among girls in the analysis stratified by sex. A meta-analysis^6^ with a large sample of children and adolescents also supported an association between victimization and sedentary behavior. It is possible that those who are bullied choose to be more reserved to decrease exposure to uncomfortable situations. Environments with less exposure to bullying, such as staying at home, may encourage sedentary behaviors including time sitting or lying down. The lack of such an association for boys in the present study may be due to the fact that boys are generally more active and perhaps prefer to adopt other activities rather than sedentary behavior.

Although bullying was related to an increase in the adoption of HRBs, that was not the case for physical activity. In fact, the analysis carried out by combining both sexes showed a positive association between victimization and physical activity. When stratified by sex, only boys who were victims of bullying practiced more physical activity. Similar to this study, a positive association between victimization and physical activity has been previously demonstrated,^29^ but there have also been studies suggesting a negative association^6^ or a lack of association.^30^ This divergence indicates that further studies on the topic are needed. The positive association for boys can perhaps be explained by the fact that those who practice physical activity are more exposed to their peers and consequently to bullying.^30^ Physical activity environments tend to have less surveillance from adults, as such sport environments may be a “trigger” for bullying actions.^29^ Besides, being more active than girls during adolescence may predispose boys to bullying victimization.^30^

The study is not without limitations. Although the sample covered participants in all economic groups, this study selected participants only from public education institutions. The self-report measures used to collect data in this study may underestimate or overestimate the study results. To minimize this risk, questionnaires were carefully selected to have extensive validity evidence to measure adolescents. As a limitation, it is also necessary to mention that data collection took place shortly after schools returned to in-person classes after a long period of social isolation caused by the COVID-19 pandemic. This may have affected some of the adolescents’ behaviors beyond what would have happened pre-pandemic.

There are strengths to this study as well. The sample was representative of the population in the city of Maringá (PR). In addition, this work contributes to the accumulation of scientific evidence examining the relationship between bullying and health risk factors. This literature is particularly scarce in Brazil. Findings from this study may help guide public policy to reduce bullying during adolescence.

In conclusion, bullying victimization was associated with an increased predisposition to the adoption of HRBs. Interestingly, victims were also more prone to participate in physical activity.

## Data Availability

The database that originated the article is available with the corresponding author.
